# Electrical wave generation and spatial organization in uterine tissue

**DOI:** 10.1098/rsif.2024.0638

**Published:** 2025-05-14

**Authors:** Shawn A. Means, Jagir R. Hussan, Amy S. Garrett, Leo K. Cheng, Alys Rachel Clark

**Affiliations:** ^1^ Auckland Bioengineering Institute, University of Auckland, Auckland, New Zealand

**Keywords:** uterus, smooth muscle, contraction, gap junctions, mathematical model

## Abstract

Healthy uterine function requires coordinated and spatially organized contractions over the menstrual cycle (oestrus in animals) and at term in pregnancy. The underlying mechanisms triggering and coordinating uterine contractions, without a distinct pacemaking region, are poorly understood. Potentially, gap-junction coupling between excitable smooth muscle cells themselves or between electrically passive cells (telocytes or fibroblasts) and excitable cells may be key. Here, we present a lattice-tissue model of coupled excitable and passive cells to investigate a potential mechanism of coordinated tissue contraction. Bifurcation analysis of cell pairs quantifies parameter windows exhibiting oscillatory behaviour. Within these windows, we demonstrate conditions when the magnitude and spatial distribution of coupling strengths generate electrical waves. Energy-based analysis of excitable cells provides quantification of intercellular energy differences cells required for spontaneous wave generation. Our model suggests passive cells must rest at a membrane voltage sufficiently higher than smooth muscle cells to trigger activity and that coupling between excitable and passive cells in spatially concentrated regions could influence the direction of tissue-wide electrical waves. This suggests that both the total number of gap junctions and their spatial expression may play a role in coordinating uterine contractility.

## 1. Introduction

**Figure 1.**
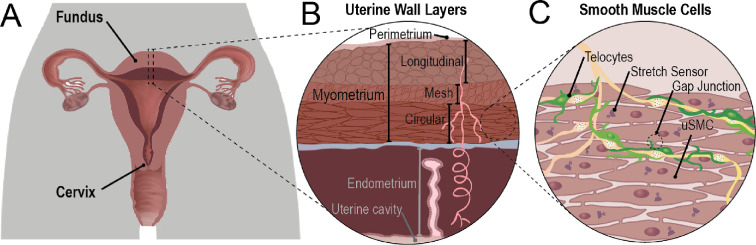
Uterine anatomy and muscle tissue composition. The human uterus with spatially distinct fundus and cervical regions (A), with magnified views of the tissue layers (B), including the muscular myometrium, and the variety of uterine cells (C), including smooth muscle and telocytes, also termed interstitial Cajal-like cells in the literature. Adapted from [1].

Coordinated muscle contractions in the uterus reflect the dynamic role of the organ both outside of pregnancy and in delivery at the end of pregnancy. Outside of pregnancy, uterine contractions are identified as varying in magnitude and direction with oestrus (animal) or menstrual (human) stages (figure 1A) [2–4]. Aside from menstruation, activity is mostly at relatively shallow depths of uterine muscle tissue (the myometrium; figure 1B) with low frequencies and magnitude so as to not inhibit implantation [3,5,6]. During menstruation, however, contractile strengths increase with greater frequency and are spatially organized from the fundus to the cervix [7,8]. Dysfunction of contractile activity in these phases is associated with disorders such as infertility, dysmenorrhoea or endometriosis [9,10].

Meanwhile, over the course of pregnancy—despite extensive expansion of uterine size—the uterine tissue is relatively quiescent. Electrical activity is shallower with only modest (if any) spatial organization [8,11]. Pregnancy culminates in the dramatic transformation at term of the uterus into a powerful and distinctly spatially organized contracting organ. The constellation of contractions, hormonal variations and extensive remodelling of size all contribute to the intricate and widely varied demands of reproductive health.

Variations in contractile magnitude, frequency and spatial organization according to the uterine phase themselves are driven by electrical activity propagating through the uterine tissue, but in the uterus, this coordination does not appear to be driven by a single distinct pacemaking cell type or region [12]. Therefore, how this coordination is achieved is somewhat of a mystery (as reviewed in detail by [1]).

The uterine myometrium (figure 1B) contains the contractile uterine smooth muscle cells (uSMC) that are responsible for uterine contraction. These muscle cells are connected to one another via gap junctions, which provide direct connections between cells, and so play a role in facilitating coordinated contractions in muscular organs. Observed increases in gap junction size and numbers between uterine cells towards the end of pregnancy may explain the transition from a relatively quiescent state to a contractile state for delivery [11,13–15]. These changes in gap junction expression may further underly observe variations in spatial organization of activity over oestrus phases in mice (for instance, [16]). By comparison, cardiac tissues additionally exhibit spatially distributed gap junctions—suggestive of not only signal facilitation over the organ but also the spatial organization of electrical activity [17]. Smooth muscle cells in the uterus are also connected to specialized cells called telocytes (also called interstitial Cajal-like cells; see [18]) as seen in figure 1C. In the gastrointestinal system, similar cells—the interstitial cells of Cajal—have a pacemaking role [19,20], yet uterine telocytes do not exhibit the same intrinsic pacemaking capacity [21]. Duquette *et al*. observed telocytes in pregnant rats and human uterine tissue at pre-labour pregnancy (caesarean section) with resting membrane voltage ($V_{m}$) higher than uSMC [21,22]. Combined with gap-junctional communication between cell types dramatically increasing at term, this may allow telocytes to modulate electrical activity in the uterus, despite not exhibiting any active electrical signalling [21]. Other tissues demonstrate this feature—for example, fibroblasts may depolarize coupled excitable cells by virtue of their higher resting $V_{m}$ and thus trigger electrical and contractile activity [23]. Additional spatial concentrations of gap junctions may further localize and organize uterine signalling observed over the course of oestrus (in mice [16]) or menstrual phases (in humans) outside of pregnancy.

The uterine pacemaking puzzle attracted several theoretical efforts to characterize mechanisms driving uterine contraction. This includes continuum models [24,25], cellular automata [26,27] and lattice-tissue models [28,29]. Lattice representations provide a tractable framework for the investigation of the dynamic interplay between different cell types. Such interplay may be responsible for generating, coordinating and spatially organizing uterine contractions. Sheldon *et al*. [28] used a discrete two-dimensional (2D) lattice of individual Fitzhugh–Nagumo (FN) [30] cells (a simplified Hodgkin–Huxley model—see §2.1) for investigating the effects of homogeneous or heterogeneous coupling between the excitable cells and activated the network with an FN pacemaker cell. Heterogeneity of their network increased the potential for global excitability across the network, whereas strong homogeneous coupling shut down the system. Their model did not consider the interaction between different cell types within the myometrium.

Xu *et al*. [29] explored the possible role of uterine telocytes using a lattice model of excitable cells, connected to a random number of passive cells (telocytes). The study employed a comprehensive uSMC model, including 19 ordinary differential equations (ODEs) and numerous additional functional evaluations for each cell [31]. The model utilized constant coupling strengths for both excitable–excitable cells (gap junctions between uSMC) and excitable–passive (uSMC and passive), in contrast to Sheldon’s approach of incorporating heterogeneity in coupling between cells. Xu *et al*. obtained results with the full uSMC model [29] that were qualitatively quite similar to prior results Singh *et al*. observed with the far simpler two-ODE FN model [32]. Synchronized oscillations emerged in local or clustered regions that eventually cohere globally given high enough coupling strengths. Some spatial patterns emerged—such as spiral waves over the lattice—but their study did not investigate potential mechanisms generating the distinct orientations observed in the uterine system.

In this study, we construct a theoretical 2D uterine tissue lattice composed of two cell types: excitable (uSMC) and passive (uterine telocytes or potentially fibroblasts that are present in the uterus). We use this model to investigate the impact of gap junctions in the generation of spatially organized signals as well as conditions for cellular excitation and oscillatory behaviour. We determine an energetic characterization of excitable cells and analyse the influence of intercellular energy differences in spontaneous wave generation. A bifurcation analysis of the underlying equations for an excitable–passive cell pair also provides known regimes of oscillations, informing the lattice-based simulations. We hypothesize (i) that differences in potential energy levels between the excitable elements in the model lattice determine the spontaneous wave initiation and propagation behaviour, and (ii) that spatial organization of these coupling strengths could influence origin and orientation of electrical waves in the myometrial tissue.

## 2. Methods

**Figure 2.**
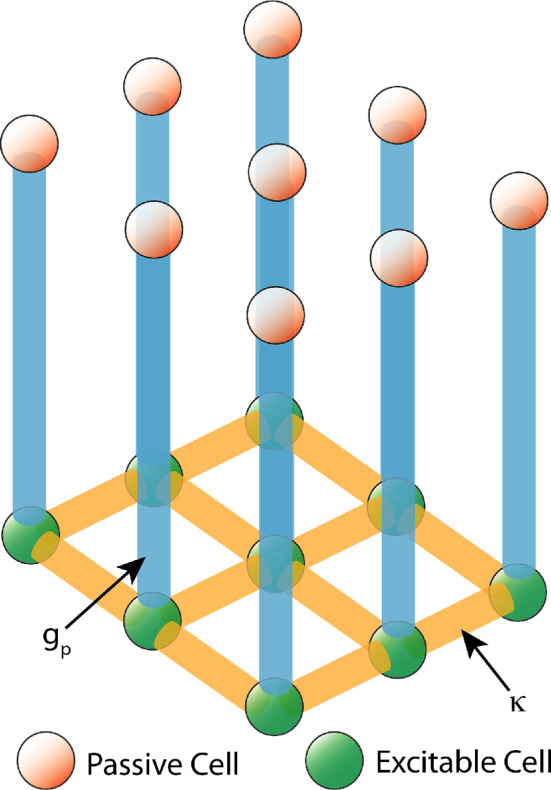
Depiction of the two layers of lattices used. Each passive cell (red sphere) is connected to a single excitable cell (green sphere) via coupling strength ($g_{p}$, blue edge). Excitable cells are further coupled to each other (yellow edges) at a given strength of κ.

We present models of electrical signals in excitable cells arranged in a square lattice and connected via gap junctions, combined with passive cells that connect to an individual excitable cell via gap junctions. This lattice is depicted schematically in figure 2.

### 2.1. Cell level models

To model excitable cells (uSMC), we use an analytically tractable excitable cell model—the FN [30]. The FN, a simplified representation for the more complex Hodgkin–Huxley model, provides not only a readily accessible mathematical formulation but also captures the overall behaviour of excitable cells with an idealized (i.e. non-dimensional) membrane voltage. Others, including ourselves, have used full Hodgkin–Huxley electrical models to represent uSMC [31,33]. However, as the aim of this study is scaling to the tissue level, we adopt the FN for a simple yet reasonable representation of excitable cellular behaviour. The FN provides a two-ODE formulation for an excitable cell (equation (2.1a)), including the idealized voltage variable, v, and recovery variable, w (see [34] for more detail). This excitable cell is then combined with a non-excitable or passive cell (telocyte or fibroblast), p (equation(2.1b)) that simply relaxes to some given resting membrane voltage $v_{\text{pr}}$


(2.1a){dvi,jdt=1ϵ[A×vi,j(1−vi,j)(vi,j−α)−wi,j−w0+Iapp−Gp−Gv]dwi,jdt=vi,j−γwi,j−v0

(2.1b)dpi,jdt=gr(vpr−pi,j)+Gp,

where subscripts i,j here indicates for the $(i,j{)}^{\text{th}}$ cell in a lattice model. The standard FN parameters as detailed in [34], A, ϵ, α and γ and the resting levels at $v_{0}$ and $w_{0}$ are set as in [28] for an excitable—but not intrinsically active—cell (table 1). As described in Sheldon *et al*., these parameters were chosen to represent excitable smooth muscle cells in the murine uterus [28]. The traditional applied stimulus term, $I_{\text{app}}$, is effectively zero throughout isolating activation through coupling with passive cells, except where explicitly stated. The relaxation rate of the passive cell to $v_{\text{pr}}$ is given by $g_{r}$, whose values were determined as described in §3.1.

**Table 1.** FN/passive cell parameters. The standard FN parameters (A, α , etc.) are taken from Sheldon *et al*. [28] for a non-oscillatory yet excitable cell representative of a uSMC. Passive cell parameters such as resting membrane ‘voltage’ ($v_{\text{pr}}$) and relaxation rate to resting ($g_{r}$) are as determined in the bifurcation analysis (§3.1). Coupling strengths κ and $g_{p}$ are drawn from normal distributions and spatially varied across the lattice as noted in the text.

parameter	A	α	γ	$v_{0}$	$w_{0}$	ϵ	$v_{\text{pr}}$	$g_{r}$	κ	$g_{p}$
value	3.0	3.0	0.05	0.4	0.4	0.2	3.0	20.0	normally distributed	
source	[28]						bifurcation analysis		spatially varied	

Coupling occurs between excitable and passive cell models (figure 2), represented through the terms $G_{v}$ and $G_{p}$


(2.2)Gv=κi−1,j(vi−1,j−v)+κi+1,j(vi+1,j−v)+κi,j−1(vi,j−1−v)+κi,j+1(vi,j+1−v)(2.3)Gp=gp(pi,j−vi,j),

where $G_{v}$ connects each excitable cell at location (i,j) with its neighbours and scaling of coupling between the excitable cells is by $\kappa_{i},j$ (yellow edges in figure 2). $G_{p}$ connects excitable to passive cells and is a simple difference between the membrane voltages in v and p scaled by a coupling term, $g_{p}$ (blue edges in figure 2). Excitation of the FN cells in our network simulations is by virtue of the depolarizing influence exerted through $g_{p}$. No other stimulus, external or intrinsic, is used.

### 2.2. Bifurcation analysis on excitable–passive cell pairs

Before simulating the lattice-tissue network, we analyse the governing equations (2.1a) and (2.1b) to assess under which parameter regimes an excitable–passive cell pair oscillates, independent of the network. This facilitates interrogating the emergent whole-network behaviour and ensures parameter regimes utilized in network simulations can produce oscillatory behaviour. A continuation package for MATLAB, MatCONT [35], provided the numerical means for finding the oscillatory regimes as determined by the key parameters $g_{p}$ (passive–excitable coupling), $g_{r}$ (relaxation rate of p) and $v_{\text{pr}}$ (resting level of p). We denoted $g_{p}$ values resulting in oscillations for our system as the range $\left[g_{\theta },\bar{g_{\theta }}\right]$ (see §3.1).

### 2.3. Defining cell–cell coupling parameters in a lattice network

For tissue scale simulations, we represent the uterine tissue as two idealized 2D lattices: one lattice of excitable cells, and the other a lattice of passive cells. The excitable lattice is connected to the passive lattice in a one-to-one fashion: each passive cell is attached to a single excitable cell at a given $g_{p}$ (figure 2). Each square lattice is sized 50 × 50 cells for a total of 5000 excitable and passive cells altogether. We can compute a rough analogous spatial extent for our lattice with actual tissue based on uSMC sizes that range from a typical 20 µm (non-pregnant) to 500 µm (pregnant, human [36]). This suggests our lattice of 50 cells corresponds to a range of about 1.0 to 25 mm, depending on phase, pregnancy and species. Pilot simulations for confirming bifurcation analysis results utilized small 5 × 5 cell ‘networks’ (see §3.1).

We utilize a heterogeneous distribution of coupling strengths ($g_{p}$ and κ) across the network. This is inspired by the report of [28] that homogeneous distributions inhibit activity and observations of spatial heterogeneity in cardiac tissues [17]. This heterogeneity is generated with normal distributions utilizing means (μ) and s.d. (σ) as distribution parameters for κ and $g_{p}$, respectively. Variations of these distribution parameters over the given ranges are noted in §3. Coupling values were often selected such that the mean ($\mu_{\text{gp}}$) straddles the values determined to produce oscillations for the coupled excitable–passive pair, $g_{\theta }$, hence producing both oscillatory and non-oscillating pairs of cells connected between the two networks.

**Figure 3.**
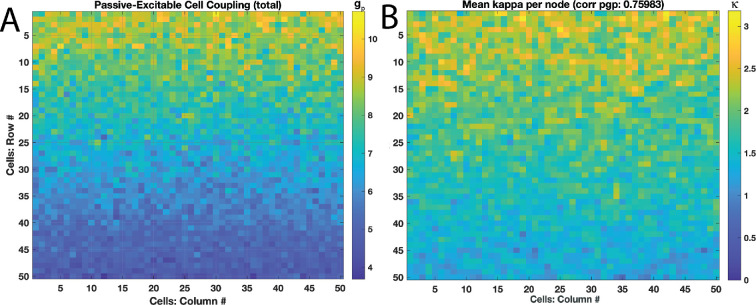
Illustration of linear spatially distributed values for coupling strengths either between passive and excitable cells with $g_{p}$ (A) or between excitable cells with κ (B). (A) shows a ‘steeply’ linear distribution with a stronger relative change in values between peaks at top border to the lower for $g_{p}$, and (B) shows a ‘modest’ linear distribution. Note that κ values plotted are average of values with neighbour cells, and correlation between $g_{p}$ and κ presented for particular realization shown (ρ=0.76). Lattice upper and lower boundaries roughly correspond to the fundus and cervix, respectively.

We applied some spatial distributions to $g_{p}$ and κ according to either a uniform or linear gradient (figure 3). For the linear gradient, we computed a planar surface of coupling strength modifier factors with a peak value at one end of the lattice and minimum at the other according to a set slope. Two variations of these set slopes or gradients (moderate and steep) are utilized for a modest or strong concentration of coupling strengths at one boundary.

### 2.4. Boundary conditions

Boundary conditions were selected to represent a tubular tissue structure, providing a simplified tubular approximation of most of a rat uterine horn (figure 1A), with rough spatial extent ranging from 1.0 to 25 mm along an edge depending on pregnancy state and species (see §2.3). Zero flux (i.e. insulating) boundary conditions were applied at the top and bottom of the lattice, aimed at correspondence with the fundus and cervix, respectively, whereas the vertical edges on the left and right were matched giving periodic or wrapped boundary conditions. To assess the impact of boundary conditions, simulations were also performed with both fully insulating boundary conditions at all edges and with the vertical edges wrapped and are noted as such in the text.

### 2.5. Numerical methods

Our system of equations resulting from equations (2.1a) and (2.1b) is a trio of ODEs per individual cell (two for the FN excitable cell and one for the passive cell), solved on a network of 50×50 cells. Numerical solutions utilized MATLAB (ode23s) running on the high-performance parallel computational platform, the New Zealand eScience Infrastructure (www.nesi.org.nz). For ease of solution and computing the influence of coupling on the system, we converted the lattice arrays into adjacency matrices analogous to those used in graph connectivities (i.e. [37]). The lattice array (equation (2.4)),  L, indicates the spatial location and neighbouring cells, and the adjacency array (equation (2.5)),  A, simplifies the aggregation of contributing factors from neighbouring and coupled cells for the ODE solver,


(2.4) $$\begin{array}{l} \text{L}=\begin{array}{l} \begin{array}{ccc} & & n_{1}\;\;\;n_{2}\;\;\;n_{3} \end{array}\\ \begin{array}{c} m_{1}\\ m_{2}\\ m_{3} \end{array}\left[\begin{array}{ccc} 1 & 4 & 7\\ 2 & 5 & 8\\ 3 & 6 & 9 \end{array}\right] \end{array} \end{array}$$

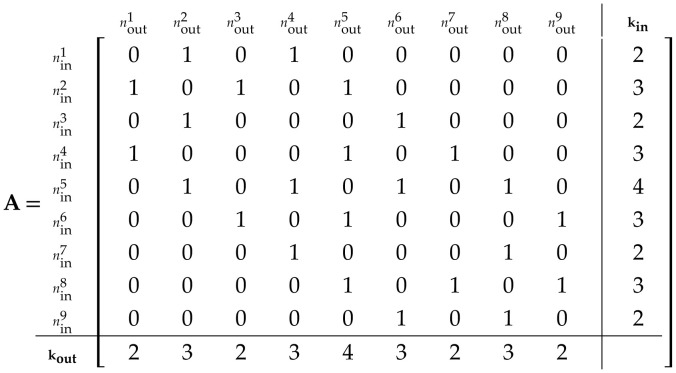



(2.5)

where $k_{\text{in}}$ and $k_{\text{out}}$ indicate the node degree of each cell in the lattice or simply the number of connections in the network for that particular cell. For instance, in the lattice, only cell 5 does not border the edge and it is thus the only cell with a connectivity of four—both inbound ($k_{\text{in}}$) and outbound ($k_{\text{out}}$). All other cells in this example lattice are edge cells with either degree of three or two at the corners.

The illustrated configuration for connectivity here in equations (2.4) and (2.5) imposes insulation boundary conditions on the lattice. We vary these to wrap the vertical boundaries by coupling the edge columns of cells. Weightings in A correspond to the coupling strengths drawn from our random distributions, be they either couplings between excitable cells ($A_{\kappa }$) or between excitable and passive cells ($A_{\text{gp}}$) as noted in equations (2.1a) and (2.1b).

### 2.6. Analysis of lattice behaviours

The aim of the lattice model is to analyse the emergence of coherent waves that propagate through the lattice network and the impact of cell–cell coupling locally on the emergence of these waves globally. To analyse wave propagation, we calculated dimensionless wave speeds (Δcells/Δt) by selecting a circular group of cells based on whether their respective v exceeded a threshold (here $v_{\text{thresh}}=2.0$). Chosen based on the first cell to rise above $v_{\text{thresh}}$, the central cell then determined the circle of cells (at radial distance of 30 cells), and we computed the average time for the wave to traverse from the central to the group of cells on the circle.

To determine whether spatial clustering of oscillatory pairs influenced the emergence of waves at the global scale, we assessed passive–excitable cell pairs, partitioned according to $g_{p}\in \left[g_{\theta },\bar{g_{\theta }}\right]$. As an estimate of overall coherency across the clusters, we deploy a variation on the Kuramoto order parameter, R,


(2.6) $$R(t)=\frac{1}{N}\sum_{j=1}^{N}e^{i\theta_{j}}.$$

For N oscillators at a given angle θ around a unit circle, |R| indicates either full asynchrony (|R|=0) or synchrony (|R|=1; see [37], for instance). We do not utilize the angular component to analyse lattices numerically since our oscillators do not rotate along the unit circle—unlike the theta-neurons presented in [37]; however, for energy-based analysis (§2.7), the angular component is used.

### 2.7. Energy-based analysis

To investigate the energy difference between the resting and average state, we follow the approach proposed by Sarasola *et al*. [38] to determine energy-like functions for well-known chaotic systems. This method applies to a dynamical system $\dot{x}=f(x)$, where f(x) is a smooth function, and relies on the ability to decompose f(x) into two components—conservative ($f_{c}$) and dissipative ($f_{d}$). Given such a decomposition, the Hamiltonian function H(x) can be calculated using the conservative field through the partial differential equation $\nabla H^{T}f_{c}(x)=0$.

This methodology enabled us to determine the Hamiltonian directly from the system of equations (equation (2.1a)), rather than deriving an energy-like function that governed the dynamics. The energy-based analysis aims to investigate the conditions for excitation propagation. Consequently, the excitable cells alone are considered since they sustain excitation propagation. The passive cells contribute towards the dissipation of the lattice energy, and their contribution is captured through $I_{\text{app}}$. For this analysis alone, $I_{\text{app}}$ is allowed to be non-zero, providing an input current to the excitable cells.

The dynamics of the excitable cell is given by equation (2.1a), omitting the passive cells in equation (2.1b). Its Hamiltonian satisfies


(2.7) $$\frac{\left(I_{\text{app}}-G_{v}^{\prime }-pG_{p}-w_{0}-w\right)}{\epsilon }\frac{\nabla H}{v}+(v-v_{0})\frac{\nabla H}{w}=0,$$

where $G_{v}^{{,}^{\prime }}$ represents the contribution from neighbouring excitable cells, $\kappa_{i-1,j}v_{i-1,j}+\kappa_{i+1,j}v_{i+1,j}+\kappa_{i,j-1}v_{i,j-1}+\kappa_{i,j+1}v_{i,j+1}.$

Equation (2.7) has the following polynomial solution for H,


(2.8) $$H=\frac{1}{2}\left(\frac{(I_{\text{app}}-G_{v}^{\prime }-pG_{p}-w_{0}-w{)}^{2}}{\epsilon }+(v-v_{0}{)}^{2}\right).$$

The excitable cell’s Hamiltonian characterizes its potential energy. Equation (2.8) enables us to calculate the potential energy given the state values and $I_{\text{app}}$. We can then construct the potential energy field associated with the excitable cell lattice and the nature of energy flows due to cell–cell coupling. This allows analysis of whether there is a difference in the potential energy levels among cells in lattices that oscillate versus those that are quiescent.

For this analysis, the excitable cells alone are considered, and $I_{\text{app}}$ is allowed to be non-zero, providing an input current to the excitable cells. The dynamics for a 50×50 cell lattice with fully insulating boundary conditions was solved numerically for 10 000, steps by which time the systems reached a steady state of either oscillating or quiescent (no-wave) status. The Hamiltonian, equation (2.8), for each excitable lattice cell based on their state values were computed over the simulated steps. Ten lattice configurations with coupling strengths from a normal distribution of μ=4.0, $\mu_{\text{gp}}=4.5$ and σ=1.0 were analysed. The resting Hamiltonian energy (the minimum Hamiltonian value) and the average Hamiltonian energy value for each cell were then calculated for each configuration. These values were then composed into 50×50 dimensional vector comprising of these values. The values were min–max normalized to help with comparison across configurations. The $L_{2}$-norm of these vectors was calculated to quantify the separation between the resting and average energy states of these lattices.

Further, we calculated the time derivative of the Hamiltonian for each cell and used the arctangent of the derivative as the order parameter θ to determine the Kuramoto coherence, equation (2.6), for the lattice sites across the various coupling weight configurations. Note that this is distinct from the numerical investigation given above using normalized v values and the norm of R, or Z=|R|.

The dynamics of the lattice is strongly governed by the coupling strengths between excitable cells and passive cells, and we investigated the parameter space defined by the energy contributions from the neighbouring cells. At the cell level, as encoded in the Hamiltonian, equation (2.8), this influence is captured by the terms $G_{v}$ and p. Note that $G_{v}$ contains currents flowing in/out from the cell’s excitable neighbours due to their membrane potential differences. Towards this, we determined the extent of $G_{v}$ and p across all the simulations (quiescent and oscillating). Using these intervals, we generated a uniform mesh grid of 500×500 points, where each point corresponds to a unique $G_{v}$ and p value within their respective intervals. We then determined whether the parameter point exists in the systems phase space (within a tolerance from the coordinate) of the simulated configurations. If the point existed in a simulated configurations phase space, we recorded the Hamiltonian energy at that phase space point. When the point exists across more than one simulated configuration, the average value of the Hamiltonian across these configurations was recorded.

## 3. Results

### 3.1. Bifurcation analysis

**Figure 4.**
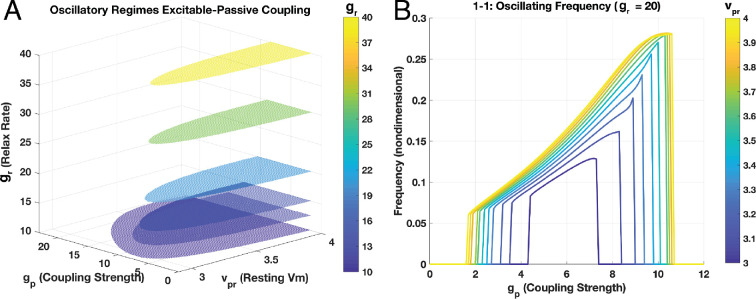
(A) Range of Hopf bifurcations indicating oscillatory regimes for a 1−1 ‘network’ (one excitable and one passive cell pair). Continuations performed over a range of passive resting levels ($v_{\text{pr}}$) and passive relaxation rates ($g_{r}$) with the passive–excitable coupling strength ($g_{p}$) being the bifurcation parameter. Oscillations occur in a restricted range of $g_{p}$ (denoted [$g_{\theta },\bar{g_{\theta }}$]). (B) Numerical scans over $v_{\text{pr}}\in [3.0,4.0]$ solving pilot networks (5 × 5 cells) for oscillating frequencies at the relaxation rate $g_{r}$= 20 confirming the bifurcation result, including the windows of oscillations with distinct lower and upper bounds for $g_{p}$. For $v_{\text{pr}}=3.0,[g_{\theta },\bar{g_{\theta }}]=[4.32,7.63]$ (dark blue trace).

Figure 4A shows the emergence of Hopf bifurcations as passive cell relaxation rate $g_{r}$, and resting voltage for the passive cells, $v_{\text{pr}}$, are varied, with excitable–passive coupling strengths, $g_{p}$, as the bifurcation parameter. The Hopf bifurcations cover a range of $g_{p}\in \left[10,40\right]$, $v_{\text{pr}}\in [2.5,4.0]$ and $g_{r}\in \left[10,40\right]$. At $g_{r}=20$, the window of oscillations closed rapidly with $v_{\text{pr}}$ around 2.96 for all values of $g_{p}$; that is, no oscillations occur for $v_{\text{pr}}>2.96$. To ensure oscillations are possible in the network, we thus utilized $g_{r}=20$ and $v_{\text{pr}}=3.0$ just above this apparent minimally oscillatory $v_{\text{pr}}$ for all subsequent simulations. For these $v_{\text{pr}}=3.0$ and $g_{r}=20$, oscillatory passive–excitable pairs occur for $g_{p}\in \left[4.32,7.63\right]$—the values of Hopf bifurcation points for these parameters (figure 4A). We denote this range $\left[g_{\theta },\bar{g_{\theta }}\right]$, providing the window of threshold values for $g_{p}$ generating oscillations.

Figure 4B shows corresponding numerical scans for frequencies of oscillations over the bifurcation parameter $g_{p}$ in pilot networks sized 5 × 5 cells without any inter-excitable cell coupling (κ=0) at the passive relaxation rate of $g_{r}$= 20. The window of oscillations is confined to the same range, $\left[g_{\theta },\bar{g_{\theta }}\right]$, as seen in the bifurcation diagram of figure 4A.

### 3.2. Heterogeneous but spatially uniform cell–cell coupling

With bifurcation analysis providing information on oscillatory regimes, the full model was then employed in a 50 × 50 array of cells. Coupling strengths $g_{p}$ and κ were drawn from normal distributions but were spatially uniform. Unless otherwise stated, excitable cells were all coupled at κ values drawn from a normal distribution with mean $\mu_{\kappa }=4.0$ and s.d. $\sigma_{\kappa }=1.0$ with n=8 realizations conducted per parameter set.

**Figure 5.**
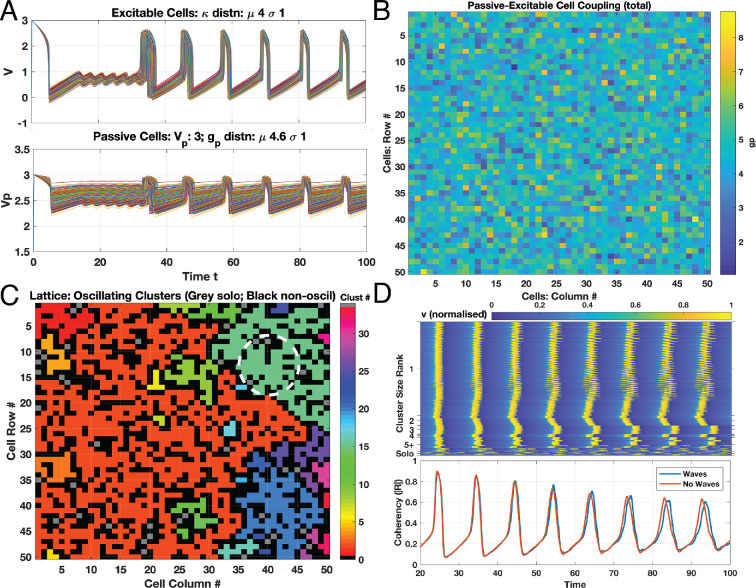
Spatially uniform coupling and waves. Passive–excitable coupling strengths ($g_{p}$) and excitable–excitable coupling (κ) are spatially uniformly distributed over the lattice of 50 × 50 cells with values drawn from normal distribution ($\mu_{\text{gp}}$: 4.6, $\sigma_{\text{gp}}$: 1.0; $\mu_{\kappa }$: 4.0, $\sigma_{\kappa }$: 1.0, all other parameters as in table 1). Oscillations over both networks emerge as shown in transient plot for all excitable (A, upper panel) and passive (lower panel) cells for this one realization (n=8) coalescing into organized spatial waves at around t=33. Spatial distribution of the coupling strengths $g_{p}$ in original simulation shown in B; note the spatially uniform distribution for values as drawn from the normal realization. Each region of known oscillating pairs (given $g_{p}\in [g_{\theta },\bar{g_{\theta }}]$) is then partitioned into clusters as shown in C, where colour indicates cluster ID for the 30+ oscillating regions (grey indicates solo oscillator; black non-oscillatory). Dashed circle indicates region of wave initiation (t=33). Simulation with same parameters and distributions as in A–C with all oscillating clusters decoupled (κ=0 at inter-cluster boundaries—black regions) and transient progress for oscillators only shown in D (upper panel, normalized v). Clusters presented according to size: largest with over 1000 cells (large orange cluster in C) down to solo (grey cells). Discordant intrinsic cluster oscillations result in coherencies as shown in D lower panel, for both simulations presenting waves (blue trace) and not (red trace). Time truncated to t>20 for excluding initial recovery from ICs with both panels at same time scale.

Considering $g_{p}$ with $\mu_{\text{gp}}=4.5$, $\sigma_{\text{gp}}=1.0$ and fully insulating boundary conditions, we observed only low amplitude and discordant oscillations despite numerous individual excitable–passive couplings at $g_{p}>g_{\theta }∼4.32$ (not shown). By increasing $\mu_{\text{gp}}$ slightly to 4.6, half of the n=8 realizations presented spatial waves. Figure 5A shows a realization where generated waves occur both with insulating and horizontally wrapped boundary conditions, with an organized spatial wave at approximately t=33. Times to wave emergence varied, but typically networks exhibit low-amplitude oscillations coalescing into a wave with eventual tight synchronization across the lattice (figure 5A). Dimensionless wave speed changes during simulation illustrate time courses of coalescence from discordant to coherent oscillations and hence waves. Initial average speeds of around 25 Δc/Δt rose to nearly 100 Δc/Δt along with the tighter spatio-temporal synchronization (see electronic supplementary material, video S1).

We assessed the distribution of $g_{p}$ between realizations, confirming there were no spatially clustered regions with high values of $g_{p}$ (figure 5B). In some realizations, waves were triggered around locations of higher $g_{p}$ values (n=10), but in other realizations with similar distributions and comparable $g_{p}$ peaks, waves were not formed. Potentially, this was due to proximal $g_{p}$ values outside the oscillatory range. When partitioning passive–excitable cell pairs according to $g_{p}\in \left[g_{\theta },\bar{g_{\theta }}\right]$ (figure 5C), several clusters and solo oscillators emerged. Two approaches were considered to assess the effect of this clustering: simulating with excitable cell–cell coupling (κ) set to zero at cluster boundaries (isolating clusters), and shuffling the spatial distribution of $g_{p}$.

Isolating clusters: intrinsic oscillations and cluster phases are now more apparent (figure 5C,D, upper panel). Native phase discordance across the network of cells emerged after initial synchrony—particularly the solo oscillators (lower panel of figure 5D)—whereas a wave coalesces in the largest cluster. However, the spatial wave emerging in the whole connected lattice does not initiate in this large cluster. Notably, isolated clusters oscillated significantly sooner than in the fully coupled lattice (t∼13) with larger clusters oscillating later at t∼24 (not shown at time scale). Asynchronous oscillators must apparently overcome intrinsic discordance prior to wave emergence, here at t∼33.

Shuffling $g_{p}$: utilizing the *exact* same realization of $g_{p}$ but spatially shuffling $g_{p}$ destroyed the wave formation in about half of n=8 simulations (see electronic supplementary material, ‘shuffling’ movies). This is despite similar sizes and distributions of oscillating pairs throughout the shuffled lattices. Isolating the intrinsically oscillating clusters demonstrated they indeed oscillate, all at independent frequencies with wave formations in the larger clusters (see electronic supplementary material, video S2). However, connecting the clusters produces only low-amplitude oscillations and no waves (not shown).

Considering the Kuramoto order parameter, R (equation (2.6)), beyond initial transient behaviour at t>20 allowed comparison of model configurations that do or do not produce waves. However, these configurations show quite similar coherencies (figure 5D, lower panel), suggesting irrelevance for wave formation. Correlations between κ and $g_{p}$ also do not explain spatial wave emergence and are neutral for all realizations regardless of wave formation (ρ∼0.02). Autocorrelations for the $g_{p}$ remain nominal with ρ∼0.09, and including weighted distance across the lattice (i.e. 1/κ) also produces neutral ρ. No correlations emerge between κ or $g_{p}$ in wave-producing distributions (maximum ρ∼0.02).

Instead of completely isolating oscillating clusters, we assessed the impact of altering κ values at interfaces between clusters (black regions in figure 5C). We considered multiplying κ values by [0.1, 0.25, 0.5 , 0.75 and 2.0] times their original value. With κ at 0.1 times its original value (with fully insulating boundary conditions), we restored spatial waves to the system (electronic supplementary material, video S3), but at lower wave speeds (mean ∼12Δc/Δt). Wavefronts were also coarser and progressed irregularly across the lattice. Doubling κ restored global waves in n=10/10 realizations with average speeds that reached up to ∼50Δc/Δt (see electronic supplementary material, video S4). Coherencies of these modified interface κ strengths produce high overall |R|∼0.8 (for 2×κ) or low |R|∼0.5 (for 0.1×κ). Interim κ fractions ([0.25,0.5,0.75]) meanwhile produced oscillating |R| varying around 0.45—reflecting the low-amplitude oscillations across the lattice failing to form a wave (see electronic supplementary material, ‘interim’ movies).

Overall, with spatially uniform distributions of the coupling parameter $g_{p}$, our model predicts global waves without any particularly striking intrinsic coherency across the network. Correlations between coupling strengths—either with $g_{p}$ and κ, weighted $g_{p}$ or autocorrelations—do not appear relevant to wave generations either. Although we can restore waves by targeting inter-cluster connectivity of κ, it is unclear at this point precisely what conditions are needed overall for wave production with this uniformly spatially distributed coupling. Hence, we next consider an analysis of the energy inputs to the system to determine whether the emergence of waves can be predicted as a function of energy considerations.

#### 3.2.1. Energy balance-based analysis

Of the ten lattice configurations analysed, three did not result in global wave propagation, and the other seven configurations resulted in spontaneous wave propagation. The energy-based Kuramoto order parameter and the $L_{2}$-norm for each lattice configuration are given in table 2. There is a clear separation between configurations in which waves do not emerge and configurations in which they do. In configurations that do not produce waves, there is a high coherence value (0.96) suggesting that all the excitable lattice elements are locked in and are at equilibrium despite exchanges of energy among neighbouring cells due to relatively low differences in energy levels. The configurations that do produce waves have a lower coherence value (0.85−0.88), indicating that spontaneous excitation induces wave propagation and synchronizes neighbouring cells. The $L_{2}$-norm suggests that resting and mean energy levels of the configurations that do not generate waves are closer to each other than that of the configurations in which waves emerge. The normalized distance between quiescent cells and oscillating cells is 0.5 (table 2), which in model energy units is equivalent to ΔH=1.41 energy units per cell.

**Table 2.** Kuramoto order parameter |R| and $L_{2}$-norm between resting and mean state of the lattice configurations.

expt no	steady-state dynamics	*R*	$L_{2}$-norm
1	no wave	0.963	7.065
2	no wave	0.963	7.067
3	no wave	0.963	7.065
4	wave	0.851	7.599
5	wave	0.851	7.599
6	wave	0.851	7.599
7	wave	0.851	7.599
8	wave	0.851	7.599
9	wave	0.851	7.599
10	wave	0.882	7.447

**Figure 6.**
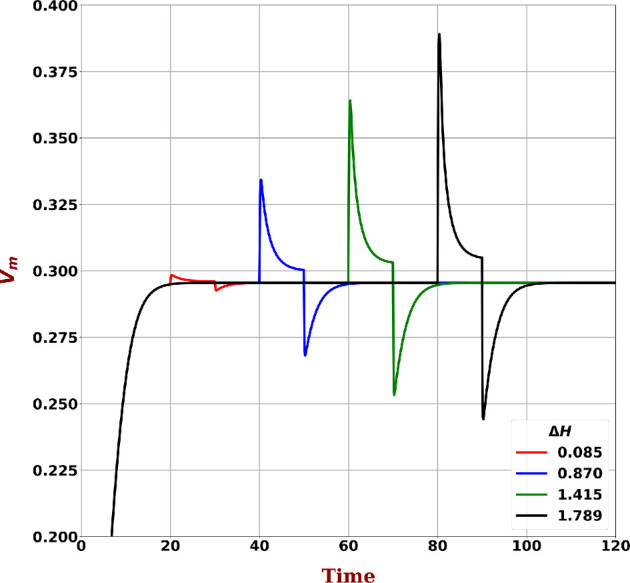
Membrane potential profiles for a single excitable cell to externally applied currents inducing energy differences of ΔH=[0.084,0.869,1.415,1.789] units are plotted. The currents are applied for 10 time units. To enable comparison, the currents were applied at 20, 40, 60 and 80 units of time after the system reached steady state.

To investigate the impact of this energy difference on an isolated lattice cell, we set p, $G_{p}$ and $G_{v}$ terms to zero in equation (2.1a) and change $I_{\text{app}}$ to replicate an increasing ΔH=[0.084,0.869,1.415,1.789] energy units. To highlight and compare the change in membrane potential, the above stimuli were applied at 20, 40, 60 and 80 units of time, respectively, with solutions to equation (2.1a) shown in figure 6. The results suggest that if a lattice configuration maintains sufficient energy difference across the lattice cells, spontaneous wave-propagation behaviour will be observed.

**Figure 7.**
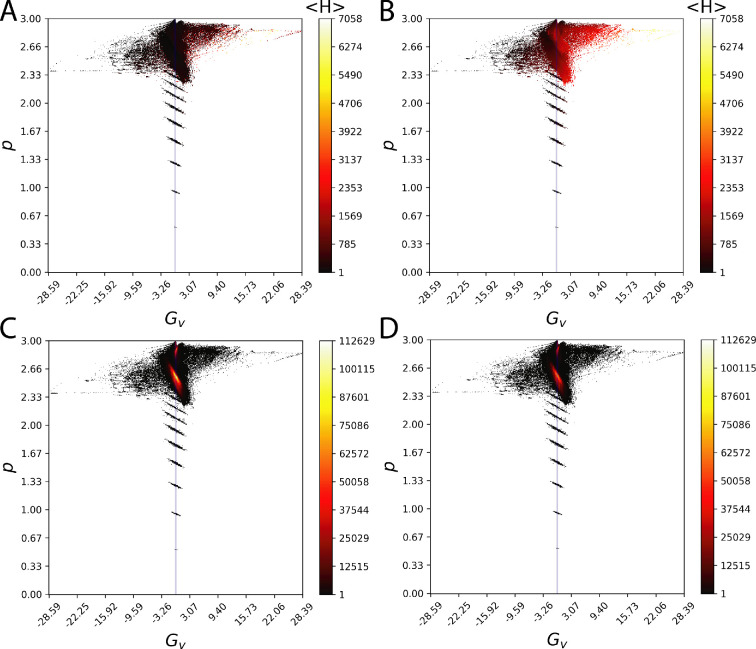
$G_{v}$ versus p space for lattice configurations that do and do not produce waves. The centre blue vertical line indicates $G_{v}=0.0$. Average Hamiltonian energies near a ($G_{v}\pm 0.01139$, p±0.00059) coordinate are shown for (A) configurations that do not produce waves and (B) configurations that do produce waves (higher energies are seen in these configurations). The number of observed instances (the number of times the coordinate was observed across all experiments) is shown in (C) configurations that do not produce waves and (D) for those that do. These plots highlight the most and least active regions of the parameter space. The energy plots suggest that the system dynamics across both non-wave forming and wave forming configurations have similar occupancy in this parameter space; however, the Hamiltonian energies are different.

Finally, we determined the Hamiltonian energy $G_{v}$ versus p space for quiescent and oscillating configurations using tolerances of ±0.01139 for $G_{v}$ and ±0.00059 for p (see figure 7A,C and B,D, respectively). The results confirm that for the quiescent configurations, the energy level is much lower (especially at the most active points figure 7A,C) than that of the oscillating configurations. Furthermore, this analysis showed that the $G_{v}$ versus p space is rather sparse and the dynamics is active around a small region of the parameters—suggesting that lattice could change its propagation behaviour by small changes to the coupling weights.

### 3.3. Spatially distributed coupling

**Figure 8.**
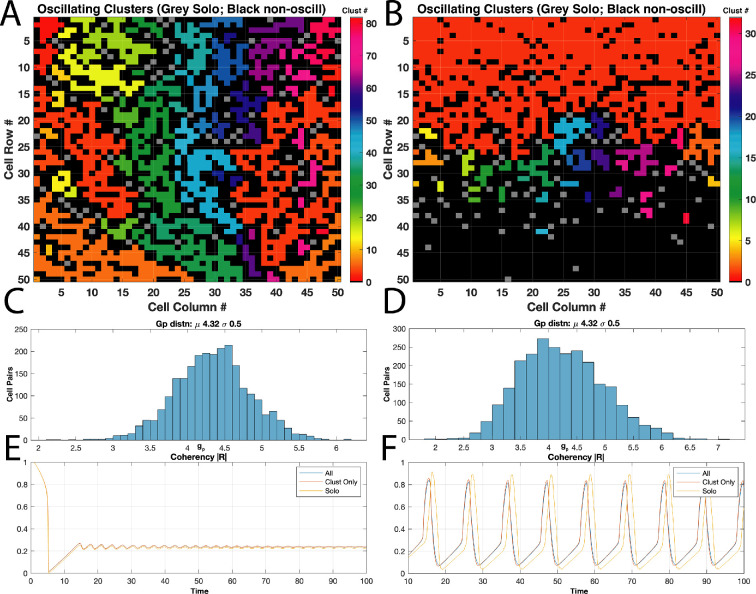
Spatial distribution impact on wave generation. Two simulations with normally distributed realizations of $g_{p}$ ($\mu_{\text{gp}}=4.32,\sigma_{\text{gp}}=0.5$) and κ ($\mu_{\kappa }=2.0,\sigma_{\kappa }=0.5$, and all other parameters the same as in table 1) but different spatial distributions. (A) Uniformly spatially distributed $g_{p}$ and κ showing resulting clusters of oscillating pairs (colours indicate cluster number; grey are solo and black non-oscillators). Percentage of oscillating pairs out of total: 52%. (B) Linearly distributed coupling strengths showing clusters of oscillators; note one large cluster dominating upper half of lattice as per linear gradient imposed (distributions shown here are from same configurations of figure 2B,C). Percentage of oscillating pairs out of total: 44%. (C,D) Histograms of $g_{p}$ values with $\mu_{\text{gp}}$ at lower bifurcation value of $g_{\theta }$ for both spatial distributions in A and B, and resulting transient coherencies for simulations utilizing shown distributions (E and F) with waves emerging on the spatially concentrated linearly distributed couplings (B).

Uniform spatially distributed couplings shown so far may produce waves but in unpredictable locations and orientations. Next, we consider spatial heterogeneity of the coupling strengths with a simple linear distribution. Aligned at either the top or bottom of the lattice boundary aimed at correspondence with the uterine fundus or cervical regions (figure 1), we distribute $g_{p}$ or κ along a plane (figure 3). Initial normally distributed realizations are conformed to this spatially linear distribution as described in §2. By virtue of the linear distribution applied to both $g_{p}$ and κ, correlations between $g_{p}$ and κ are significant unlike in the uniform cases. For instance, the cluster plot in figure 8B is the same realization as in figure 3B—modified with the linear spatial distribution—but now with ρ∼0.76.

With all other parameters identical, spatially distributed coupling strengths produce waves in every instance where the uniform distributions fail (n=5; figure 8). Wave initiation and orientation are further dictated by a dominant oscillating cluster aligned at the lattice top (red cluster, figure 8B; see electronic supplementary material, video S5). Strikingly, the proportion of total oscillators is higher in the uniformly distributed case (52% as opposed to 44%), but produces no waves. Nevertheless, linearly distributing $g_{p}$ can generate waves with insignificant values of ρ—when κ is not linearly distributed. Comparisons of uniformly and linearly distributed $g_{p}$, and uniformly distributed κ, with all other parameters the same produced fewer waves in the uniform (2 / n=5) than the distributed (5 / n=5) with ρ close to zero throughout (all O(−3), not shown).

**Figure 9.**
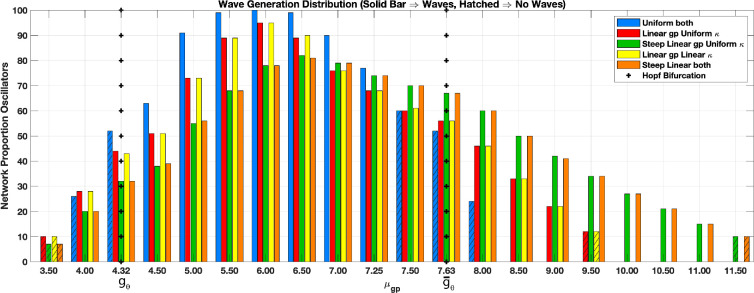
Wave generation $\mu_{\text{gp}}$ dependency. Bar chart indicates average proportion of oscillating cell pairs out of total network for suites of realizations (n=5) at given $\mu_{\text{gp}}$ for a variety of spatial distributions. Legend indicates uniform or linear distribution for $g_{p}$ and κ with two varieties of linear distribution (modest or steep gradient) and combinations thereof. Vertical ‘+’ indicates values of bifurcation, $\left[g_{\theta }=4.32,\bar{g_{\theta }}=7.63\right]$, for generating oscillations (as depicted in figure 4). Each bar with solid colour or hatching indicates whether waves or no waves result, respectively. Coupling parameters: $\mu_{\kappa }=2.0,\sigma_{\text{kappa}}=0.5$, $\sigma_{\text{gp}}=0.5,\mu_{\text{gp}}$ as plotted with horizontally wrapped BCs; all others standard as given in table 1.

Figure 9 summarizes the incidence of oscillating cell pairs and spatial waves across all simulations conducted. Over a wide range of $\mu_{\text{gp}}$, waves result with spatially concentrated coupling that does not when uniformly distributed. Within the interval of expected oscillations (i.e. $g_{p}\in \left[g_{\theta },\bar{g_{\theta }}\right]$), waves do not necessarily emerge for uniformly distributed coupling, whereas they do for all cases when linearly distributed. Outside that interval, waves persist with linearly distributed coupling for $\mu_{gp}>\bar{g_{\theta }}$. Furthermore, proportions of oscillators are again consistently lower in the linearly distributed cases, i.e. 20% producing waves instead of at least 60% for the uniform (compare blue bars for uniform with others).

Extension of wave production beyond $\bar{g_{\theta }}$ is more pronounced when both $g_{p}$ and κ are similarly spatially distributed (compare red with green and orange bars; figure 9). This is partly due to the linear concentration of $g_{p}$ resulting in values well over $\bar{g_{\theta }}$ at the upper boundary, but falling within $\left[g_{\theta },\bar{g_{\theta }}\right]$ at the lower—effectively shifting the oscillating cluster from the top to the lattice bottom. As a result, waves trigger at the lower boundary and proceed upwards (see electronic supplementary material, video S6).

Wave characteristics themselves vary considerably with wave formations dying out (electronic supplementary material, video S6) or damped (electronic supplementary material, video S7). Cusped and split wavefronts may emerge along gaps of coupling (electronic supplementary material, videos S7 and S8) or interior clusters may generate waves travelling both up and down (electronic supplementary material, video S9). Alternatively, uniform spatial distributions trigger waves at random locations (electronic supplementary material, videos S10 and S11) that may coalesce into spiral waves (electronic supplementary material, videos S12 and S13), reminiscent of circulating excitations in pregnant uteri [39].

Overall, the proportion of oscillating pairs in the lattice appears critical to wave generation, with uniform distributions requiring the highest level (approx. 60%, blue bars; figure 9). Spatially concentrating oscillators reduce this threshold considerably down to 20 and 15% for the modest and strong linear distributions, respectively (green and orange bars).

## 4. Discussion

In this study, we analysed the emergence of spatial waves in an idealized representation of uterine myometrial tissue. Our model defined via coupled 2D lattices of excitable FN cells (representing uSMC) and electrically passive cells (representing telocytes) indicates feasibility for the myometrium to generate electrical activity simply by virtue of connections between these cell types. Biologically, these connections are likely to relate to gap junctions, which provide communication channels between cells. Gap junctions are known to change over the course of oestrus–menstruation and pregnancy [13], which we represented with varying κ (uSMC–uSMC connections) and $g_{p}$ (uSMC–passive cell connections). The uSMC parameters we inherited from Sheldon *et al*. FN model [28] were derived from pregnant murine uterine tissue. However, as the FN excitable model is non-dimensional in our idealized lattice tissue, the results are applicable across species or oestrus/menstrual phases as well as pregnant or not. There is a strong dependence on the level of connectivity between the two cells represented. Hence, our focus is on the impact of electrical coupling between the cell types over strength and space.

Overall, we observe the emergence of electrical activity without any explicit pacemaking cell or extrinsic stimulation (i.e. $I_{\text{stim}}=0$) with sufficient coupling strength between disparate cells that are at sufficiently distinct resting $V_{m}$. Lacking either of these elements, it is unlikely the tissue system as we defined and simulated here will generate any activity. Moreover, the site and spatial organization of activity that does emerge is readily controlled by virtue of spatially distributing the key coupling strengths between our two cell types. By concentrating the coupling—or gap junctions—we can calibrate the initiation site and direction of electrical activity, suggestive of how uterine tissue may simply focus and direct electrical and hence contractions over different phases of the organ’s dynamic roles.

### 4.1. Spatially uniform cell–cell coupling

First, via bifurcation analysis, we established conditions under which an excitable–passive cell pair would oscillate, independently of any applied stimulus. Next, we assessed the coupling of excitable and passive cells in a 50 × 50 lattice, with uniform distributions of cell coupling. Uniform spatial distributions of cell–cell coupling may present waves with an expected dependency on the strength of coupling, particularly $g_{p}$, but oscillatory regimes for $g_{p}\in \left[g_{\theta },\bar{g_{\theta }}\right]$ are not a simple determinant of wave generation, however. For instance, no waves resulted with $\mu_{\text{gp}}=4.5$ just above $g_{\theta }=4.32$. Yet with a slight rise in $\mu_{\text{gp}}$ to 4.6, waves emerged in a number of simulations (figure 5).

Although Xu *et al*. did not perform an analytical bifurcation of conditions necessary to generate oscillations for their disparate cell models as we did here, they nevertheless demonstrated a thresholding behaviour dependent on intercellular coupling similar to our observations [29]. As they increased intercellular coupling between excitable and passive cells, gradual emergence of small clusters, then localized regions and eventual global oscillations occurred with a distinctive demarcation between no oscillations and lattice-wide (see their fig. 6, for instance). Alternatively, networks of intrinsically oscillating ‘cells’ when coupled together in particular ways—such as with theta-neurons or Winfree oscillators—can exhibit a network-wide paralysis, locking the entire system into quiescence [37,40], demonstrating the complex dependency of emergent network behaviour.

Overall, waves occurred when the proportion of oscillators throughout the lattice exceeded 60% for uniform spatial distributions. Shuffling a wave-producing distribution of $g_{p}$ may disrupt the formation of waves, but with targeted modulation of κ, these waves could be restored (figure 5C). Hence, the underlying connectivity of κ may act either as bridges propagating the wave or as barriers blocking them. This may also be the mechanism at work in the Xu *et al*. simulations, such that as the coupling is successively increased, the resistance to oscillations is overwhelmed [29]. Notably, neither coherencies (|R|) of oscillating clusters nor correlations (σ) appear important for wave production. Rather, with high enough $\mu_{\text{gp}}$, nuances disappear as proportions of oscillators exceed 60%, and all simulations reliably produce waves (figure 9). At the upper range with $\mu_{\text{gp}}$ just below $\bar{g_{\theta }}$ where the proportion is at 60%, waves then fail to emerge (figure 9, hatched blue bars).

### 4.2. Energy analysis

We further analysed a simplified system by focusing on the excitable cell energy states and found that these energy states provided a threshold for the emergence of spatially organized waves. Given sufficient difference between the resting and average levels of energy in the lattice (table 2), waves result. Since there are no external inputs into the system, this difference is generated by virtue of the passive cell influence on the excitable and distribution of resulting energy across the excitable lattice through $G_{v}$—and necessarily κ and $g_{p}$. The L2 norm differences shown in table 2 show the sensitivity to the degree of elevation for energy of the system above resting. The L2 norm provides an indication of the mean energy differences between excitable cells in the lattice. This excess energy flows through the lattice until all cells are at similar energy levels (such that their energy difference is lower than the energy barrier between the cells). We demonstrated that the mean energy difference observed in the oscillating configuration is sufficient to induce a membrane voltage depolarization in a single cell. Such depolarizations would generate a spontaneous excitation wave and contribute to exchange of energy across the lattice cells. We also showed that there is an energy-difference threshold below which no significant depolarization occurs. Thus when energy-difference within a lattice is below this threshold, no spontaneous wave propagation is initiated. For the numerical experiments conducted here, the energy flux corresponded to the mean-energy difference was injected into the single cell utilizing $I_{\text{app}}$, although it should be clear that for a cell in the lattice this energy flux will be injected through $G_{v}$ and $G_{p}$.

### 4.3. Spatial distribution influence

Spatially concentrating coupling strengths produce waves with significantly lower proportions of oscillators than required with the spatially uniform. In fact, with as few as 15% oscillating cell pairs on the lattice, waves emerge when $g_{p}$ is predominantly arrayed along the border (figure 9, green and orange bars). Aggregation of $g_{p}$ thus can reduce the overall required proportion of oscillators for waves to emerge by around a factor of 4. Correlations between the κ and $g_{p}$ coupling again appear irrelevant to wave production. κ (uSMC–uSMC connections) can either be steeply linearly distributed (high ρ∼0.7; figure 9) or simply uniform with a vanishingly low correlation (ρ∼O(−3)). $g_{p}$ is therefore of primary importance for excitation into a wave given a sufficient κ that ensures propagation of the wavefront across the lattice. Localized concentrations of $g_{p}$ further determine the origin and orientation of waves—when spatially distributed. We can dictate triggering of waves at a boundary and direct them towards the opposite end by simple aggregation of $g_{p}$ and in a scenario that otherwise fails to produce any wave (figure 8).

By contrast, lack of spatially concentrated $g_{p}$ generates uncoordinated electrical activity from rather random points of wave initiation reminiscent of numerous observations in uterine tissue where electrical waves appear ‘suddenly in random locations that were not contiguous or anatomically related to each other’ [41–45]. Due to electrical triggering of calcium influx, these multiple and uncoordinated eruptions of action potentials further manifest in chaotic calcium patterns with complex implications for contraction [16,36]. Moreover, the circular and spiral waves we obtain with our lattice model further evoke observations of circus movements in rats at the end stage of pregnancy that may correspond to shortened, premature or dysfunctional labour—as well as the experimental preparation [39].

### 4.4. Physiological implications

This study aims to illuminate potential interactions between uterine cell types for initiating and orienting electrical waves in uterine tissue. Several *in vivo* and *ex vivo* experimental approaches show coordinated waves of activity in rats [12,46,47], mice [16] and humans [10], yet no pacemaking site is identified. Establishment of coordinated electrical wave propagation without a pacemaking region appears feasible given our idealized 2D multi-cell lattice investigations. This feasibility comes with some caveats, however. Although observations in myometrial tissue of increased gap junction density prior to term are established [11,13–15], the manner of change in the number or location of gap junctions throughout oestrus or menstrual phases in animals or humans outside of pregnancy is not well known. Nevertheless, observations of gap junction variability in non-pregnant humans [48] are suggestive—particularly combined with spatially reorganized uterine signals per oestrus phase in mice [16]. Meanwhile, during pregnancy, the diminished directionality and shallow electrical activity—further evocative of some of our results here—indicate yet another distribution and strength of gap junction expression for the dynamic roles of the uterine organ [8].

Our investigations here therefore suggest several lines of inquiry for experimental confirmation, including the following. (i) Is there a non-uniform spatial distribution of gap junctions in the myometrium? (ii) Do these spatial distributions correspond to oestrus or menstrual phases and not just to pregnancy and term, i.e. alignment of gap junction concentration at the fundus during term or cervix facilitating fertilization?

In cardiac systems, gap junctions are found to express a heterogeneous spatial distribution in order to facilitate directional propagation of electrical signals from the sino-atrial node to the atrial-ventricular node [17,49]. Our results indicate that if analogous spatial distribution of gap junctions is present in the uterus, this may be key to driving coordinated contractions. Higher levels of the gap junction protein connexin-43 reported as localized to the upper segment of the pregnant uterus, albeit in cultured myometrial tissue [50], supports this but with caution on viability of cultured SMC known to lose excitability. However, in cardiac systems, the directionality of contraction is consistent across every beat in an almost identical fashion. If a gap junction distribution is implicated in directional electrical activity in the uterus, the spatial distributions may vary with respect to the hormonal cycle and pregnancy. Gap junctions have a turnover of 1.5−3 h [51], suggesting hormonal changes could feasibly drive spatially varied gap junction expression at different stages of the oestrus/menstrual cycle, over pregnancy or at term. This awaits experimental confirmation.

Of particular concern is the role of the telocyte as a passive cell in our theoretical framework. Resting membrane voltages, $V_{m}^{r}$, for the uSMC are observed ranging from −70 mV at pregnancy outset to −55 mV at term [22]. Telocytes on the other hand exhibit $V_{m}^{r}$ at around −58 ± 7 mV [21]. The difference in resting membrane potential between the two cell types is hypothesized key to uterine contractions [21–23]. However, our bifurcation analysis of the FN system in equations (2.1a) and (2.1b) indicates no oscillations occur for $v_{\text{pr}}$ lower than a non-dimensional value of approximately 2.96 (figure 4). Meanwhile, resting v for the FN excitable cells with parameters in table 1 is around 0.3611 (also non-dimensional). This suggests $v_{\text{pr}}$ must be sufficiently—and substantially—higher to excite coupled uSMC, and with the FN model used here, by around a factor of eight. Xu *et al*. modelling physiological $V_{m}$ in their lattice tissue (with a uSMC $V_{m}^{r}$ at −54 mV) observed no oscillations if the passive cells are at $V_{m}^{r}$ lower than around −42.5 mV ([29], see their figure 3). Given the observation noted above that telocytes display $V_{m}^{r}$ of*—at most*—about −51 mV, it is unlikely telocytes can excite activity in a coupled role with uSMC in the myometrium. This may change of course prior to term during pregnancy, or, particularly, in the non-pregnant scenario where data are quite sparse [1]. It may be, therefore, telocytes—given their $V_{m}^{r}$ close to or even lower than the uSMC $V_{m}^{r}$—are instead dampers of activity. Other passive cells in the tissue, such as fibroblasts with their $V_{m}^{r}$ ranging far higher up to −15 mV [52], may perform an excitatory role since the potential for depolarizing any connected uSMC above threshold for activation is more likely.

Regardless, experimental determination of spatial distributions for gap junctions in the myometrium and suitability of uterine fibroblasts as excitatory for coupled uSMC would be advantageous. Hormonal phases such as the oestrus/menstrual cycle may influence the spatial distribution of gap junctions, and therefore the coordination and directionality of electrical waves in the uterus outside or during pregnancy as well as at term. These intriguing possibilities for the generation of intrinsic myometrial electrical activity and diagnostic potential await the experimental establishment of signal generation and orientation origins.

## 5. Limitations

Our 2D spatial lattice representation of uterine tissue did not consider the varied locations of passive cells. Spatial cellular heterogeneity may be an alternative or additional influence to spatially concentrated gap junctions. Furthermore, our focus on a potential mechanism for generating uterine electrical signals did not include consideration of mechano-sensitivity [42,53]. Moreover, our use of the FN model complicates any comparison with physiological data, such as wave speeds or membrane voltages, due to its inherent non-dimensional parametrization.

Further work with the inclusion of physiologically based electrical models reflecting cell-level activity [33] will provide further insights into these mechanisms with results more comparable to experimental data, yet with complications to any bifurcation analysis. Influence of oestrus phases and hormonal variations on uSMC behaviour that may dramatically impact ion channel expressions [54,55] and thus sensitivity to any gap junction variations will call for models to include both aspects.

## 6. Conclusion

Our lattice-based myometrial tissue model including two disparate cell types, excitable (uSMC) and passive (telocytes/fibroblasts), demonstrates a feasible mechanism for the generation of uterine electrical signalling. Given sufficient resting membrane voltage differences and gap-junctional coupling, passive cells excite smooth muscle cells. Moreover, spatially organized gap junctions in the tissue spatially orient and coordinate electrical signals during dynamic phases of uterine function and are orchestrated without a distinct pacemaker region. Our model’s demonstration of electrical initiation and organization in the uterus via spatially heterogeneous gap junction distributions awaits experimental confirmation. Moreover, establishing the mechanism underlying the generation and organization of uterine electrical activity may inform clinical diagnostics for detecting abnormalities prior to term. For instance, measurements of degrees of electrical organization and spatial orientation may thus indicate the successful establishment of critically concentrated gap junction connectivities, with potential impacts for uterine dysfunction including endometriosis, adenomyosis, dysmenorrhoea and infertility to name a few. Resolving any issues well before the critical labour phase holds great potential, all contingent on illuminating the mysterious underlying mechanism of the uterus.

## Data Availability

The Physiome Model Repository contains implementations of the uSMC models published by Means *et al*. [33] (https://models.physiomeproject.org/e/b43/Tong_Choi_Kharche_Holden_Zhang_Taggart_2011_reduce1_ss1_currentmod1.cellml/view) as well as Tong *et al*. [31] (https://models.physiomeproject.org/e/263/Tong_Choi_Kharche_Holden_Zhang_Taggart_2011.cellml/view). Codes used to generate lattice results are available at the University of Auckland Figshare repository (https://figshare.com/s/2d9028a16e565222a54d). Videos of selected simulations are provided online at the University of Auckland Figshare (https://figshare.com/s/062c10f5c3d7a50b5816).
